# The Global Ecology and Epidemiology of West Nile Virus

**DOI:** 10.1155/2015/376230

**Published:** 2015-03-19

**Authors:** Caren Chancey, Andriyan Grinev, Evgeniya Volkova, Maria Rios

**Affiliations:** United States Food and Drug Administration, Center for Biologics Evaluation and Research, Silver Spring, MD 20993-0002, USA

## Abstract

Since its initial isolation in Uganda in 1937 through the present, West Nile virus (WNV) has become an important cause of human and animal disease worldwide. WNV, an enveloped virus of the genus *Flavivirus*, is naturally maintained in an enzootic cycle between birds and mosquitoes, with occasional epizootic spillover causing disease in humans and horses. The mosquito vectors for WNV are widely distributed worldwide, and the known geographic range of WNV transmission and disease has continued to increase over the past 77 years. While most human infections with WNV are asymptomatic, severe neurological disease may develop resulting in long-term sequelae or death. Surveillance and preventive measures are an ongoing need to reduce the public health impact of WNV in areas with the potential for transmission.

## 1. Introduction

First described in 1937 from a febrile illness case in Uganda, West Nile virus (WNV) caused infrequent outbreaks typically associated with mild febrile illnesses from the 1950s through the 1980s in Israel, Egypt, India, France, and South Africa [1–11]. The first outbreak of neuroinvasive disease caused by WNV (WNND) was reported among the elderly in Israel in 1957 [6, 11]. Subsequent outbreaks included adult and pediatric WNND cases [4, 5, 9, 12, 13].

**Figure 1:**
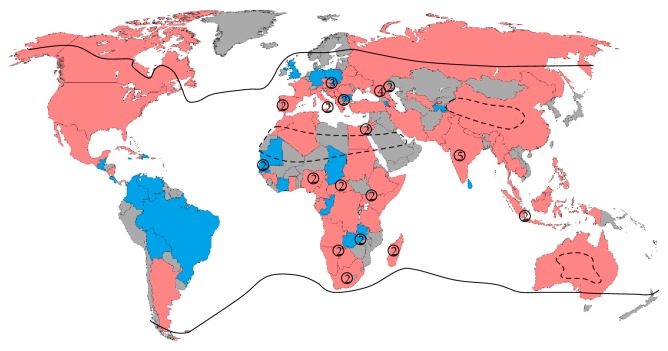
Global distribution of WNV by country: Red—human cases or human seropositivity; Blue—nonhuman/mosquito cases or seropositivity; Gray—no data or no positives reported. Black lines represent worldwide distribution of the main WNV mosquito vectors, excluding areas of extreme climate denoted by dashed lines. Circled numbers indicate the reported presence of WNV lineages other than lineage 1 in that specific area. For Japan, South Korea, Finland, and Sweden, seropositivity for WNV has been detected only in nonresident birds, which was not considered indicative of local transmission. Kading et al. [182] reported infections in gorillas living near the border of the Democratic Republic of the Congo and Rwanda, which were sampled in the D.R.C., but may have been infected in Rwanda.

Starting in the mid-1990s, the frequency, severity, and geographic range of WNV outbreaks increased, and outbreaks of WNV meningitis and encephalitis affecting primarily adults struck Bucharest, Romania, in 1996, Volgograd, Russia, in 1999, and Israel, in 2000 [14–16]. WNV crossed the Atlantic and reached the western hemisphere in the summer of 1999 when a cluster of patients with encephalitis was reported in the metropolitan area of New York City, New York, in the United States, and within 3 years the virus had spread to most of the contiguous U.S. and the neighboring countries of Canada and Mexico. In addition, although few human cases have been reported, WNV has also been found in Central and South America through surveillance studies in field specimens, suggesting a potential risk for an outbreak in humans [17, 18]. In the 77 years since its discovery, the virus has propagated to a vast region of the globe and is now considered the most important causative agent of viral encephalitis worldwide (Figure 1).

## 2. Viral Genome and Structure

**Figure 2:**
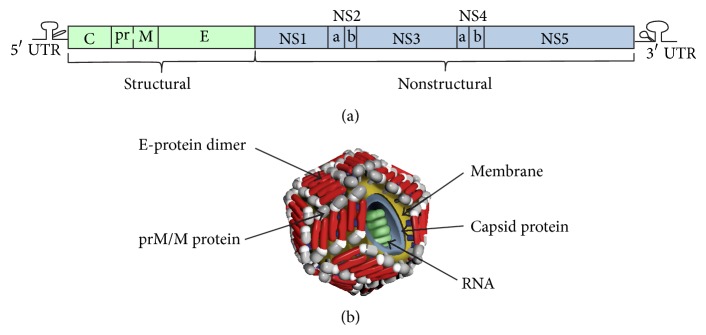
WNV genome organization and virion composition: (a) the viral genome is represented with one ORF encoding 3 structural and 7 nonstructural proteins. The 5′ and 3′ UTRs are indicated. Structural proteins are colored green, whereas nonstructural proteins are blue. (b) Structure of WNV virion.

WNV belongs to the genus* Flavivirus*, family Flaviviridae, and is a member of the Japanese encephalitis serocomplex, which also includes Japanese encephalitis virus, St. Louis encephalitis virus, Rocio virus, and Murray Valley encephalitis virus [19, 20]. Like other flaviviruses, WNV has a single-stranded positive-polarity RNA genome of approximately 11 kb, containing 10 genes flanked by 5′ and 3′ noncoding regions (NCR) with no polyadenylation tail at the 3′ end [21–25]. The NCRs of the WNV genome form stem-loop structures essential for viral replication [26, 27]. The viral genome encodes a single polyprotein that is co- and posttranslationally cleaved into 3 structural proteins: Capsid (C); Pre-M/Membrane (prM/M); and Envelope (E); and 7 nonstructural (NS) proteins: NS1; NS2A; NS2B; NS3; NS4A; NS4B; and NS5 [24, 28] (Figure 2).

Structurally the WNV virion is a ~50 nm icosahedral particle, surrounded by a lipid bilayer (reviewed in [29]). The nucleocapsid is composed of C protein, which associates with the RNA genome and mediates viral assembly [30, 31]. Heterodimers of prM and E protein become embedded in the lipid bilayer of the virus during assembly and are exposed on the virion surface [32]. The prM protein is thought to protect the immature virion from undergoing premature fusion prior to viral budding from the cell surface by blocking the fusion loop of E and is cleaved off during the viral maturation process [32–36]. During infection, mature, immature, and partially mature virus particles are produced, containing a varying number of immature prM protein molecules on the surface [37]. The E protein mediates both binding of the receptor on the cell surface for viral entry and fusion with the membrane of the host cell [38–40].

The seven nonstructural proteins are multifunctional, playing critical roles in viral RNA synthesis and/or assembly. NS1 is believed to play an early role in regulation of viral replication [41–43]. NS3 has multiple enzymatic functions, serving as a viral serine protease which cleaves the other nonstructural proteins from the viral polyprotein, in association with NS2B; an RNA helicase in association with NS4a; and an NTPase in association with NS5 [44–49]. The NS5 protein is necessary for viral replication, containing RNA-dependent RNA polymerase (RdRp) activity in the C-terminal region and methyltransferase activity in the N-terminal region [41, 50–53]. NS2A, NS2B, NS4A, and NS4B are small, hydrophobic proteins that have no known enzymatic functions, but are believed to act as cofactors for viral replication complex assembly and localization [54–57].

The WNV NS proteins can also modulate cell signaling and immune responses [58–67]. In particular, the WNV NS1 protein antagonizes the host's antiviral defenses through inhibition of TLR3 signal transduction and STAT1/STAT2 activation [64, 67]. It has also been shown that NS1 inhibits complement activation through fH and C4b binding, contributing to flavivirus immune evasion [58–60]. Alternatively, cell surface-associated NS1 represents a major target for host antibodies which contributes to clearance of WNV-infected cells through Fc-gamma receptor-mediated phagocytosis [61].

## 3. Genetic Classification

**Figure 3:**
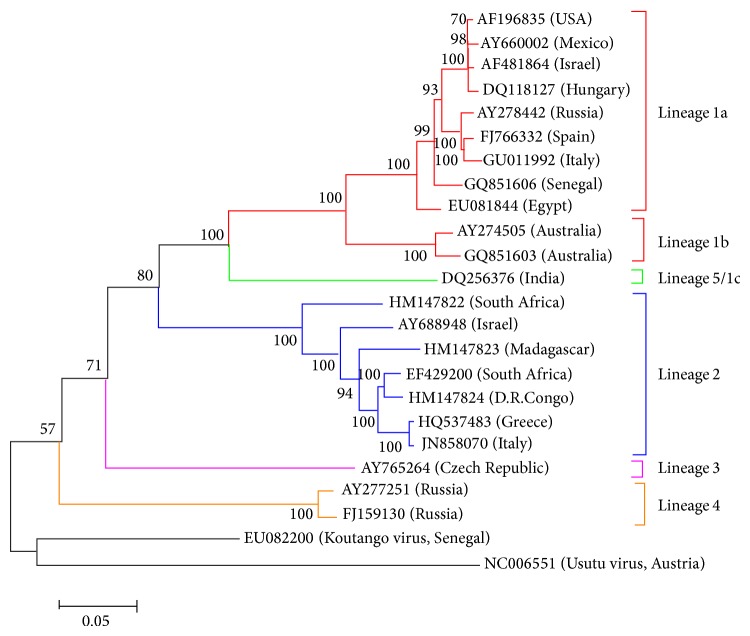
Major WNV lineages. Maximum-likelihood phylogenetic tree is based on complete genome sequences and Nearest-Neighbor-Interchange as heuristic search method. The tree was constructed using MEGA 6 with 1000 bootstrap replications. The tree was rooted using Koutango and Usutu viruses.

WNV is a genetically and geographically diverse virus. Four or five distinct WNV genetic lineages have been proposed based on phylogenetic analyses of published isolates [3, 68–73]. Their genomes differ from each other by more than 20–25% and correlate well with the geographical point of isolation (Figure 3). Lineages 1, 2, and 5 of WNV have been associated with significant outbreaks in humans [3, 68, 72, 74, 75]. Lineage 1 is distributed widely throughout the world and consists of two clades: 1a and 1b [76, 77]. Clade 1a includes isolates from Africa, Europe, the Middle East, Asia, and the Americas. Clade 1b is represented by the Australian Kunjin virus (KUNV).

Phylogeographic analysis has shown that the most probable origin of WNV lineage 1a was sub-Saharan or Northern Africa [78]. This clade emerged in the beginning of the 20th century and then spread northwards in the 1970s–80s, mainly following the eastern bird migratory route connecting Northern Africa and Israel with Russia and Central Europe. Later, in the 1990s, a strain of WNV genotype 1a appeared in Morocco and Western Europe, where the virus became endemic, causing small sporadic outbreaks. In 1999; this WNV lineage 1a virus was exported, most likely from the Middle East, to the Americas, where it spread over North America and then to South America, making WNV a global public health problem [78]. Zehender et al. further divided clade 1a into A and B subclades, with most isolates from Western Europe and some from Eastern Europe belonging to subclade A and the remaining Eastern European isolates belonging to subclade B [78].

WNV lineage 2 isolates are historically endemic in sub-Saharan Africa and Madagascar and have caused sporadic zoonotic outbreaks in South Africa [71, 76, 79]. More recently, WNV lineage 2 strains have been associated with bird and human outbreaks in southern and eastern Europe [80]. Lineage 2 WNV was also sequenced from a 2004 Indonesian clinical specimen [81]. It has been suggested that WNV lineage 2 originated in Africa and was introduced into Europe, where it became endemic, on at least two separate occasions during the last two decades [82].

Lineage 3 of WNV is represented by a pair of isolates from mosquitoes collected in the Czech Republic border region near Rabensburg, Austria, in 1997 and 1999, which have been shown experimentally to infect only mosquitoes and mosquito cells [68, 83]. Lineage 4 comprises viruses circulating in Russia since 1988, including a tick isolate from the south-west Caucasus and a number of isolates from mosquitoes and reptiles in the delta of the Volga river [84, 85]. Lineage 5, formerly considered clade 1c of lineage 1, includes isolates from India from 1955 to the present [3, 72, 86].

Other potential lineages of WNV have been described, including Koutango virus from Africa, a group of isolates from Spain, a variant of Kunjin virus isolated from Sarawak, Malaysia, and a Senegalese isolate [73, 75, 87, 88].

## 4. Hosts and Vectors

### 4.1. Hosts

**Figure 4:**
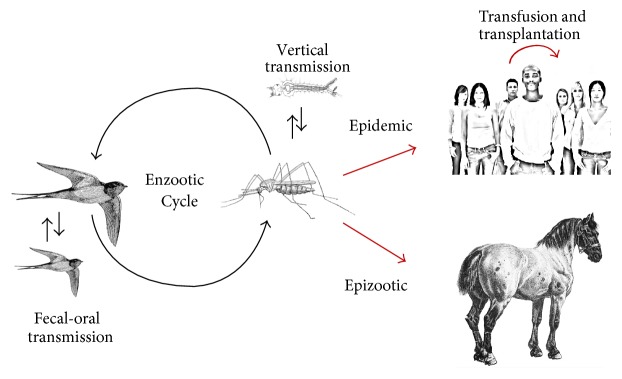
WNV transmission cycle: enzootic amplification of WNV by birds and mosquitoes supplemented by bird-to-bird transmission and transmission between cofeeding mosquitoes. Vertical transmission by mosquitoes provides the mechanism of virus overwintering. Humans and horses are counted as incidental dead-end hosts. Human-to-human transmission may come through blood transfusion, organ transplantation, and breast feeding and in utero.

Maintained in nature in an enzootic transmission cycle between birds and mosquitoes, WNV can also infect humans and other vertebrates and cause serious disease and death (Figure 4). Birds are considered the most important hosts for the WNV life cycle because they can develop viremia sufficiently high to infect mosquitoes (reviewed in [89]). Birds in the family Corvidae such as American crows (*Corvus brachyrhynchos*) and blue jays (*Cyanocitta cristata*) become ill or die from WNV, but other birds such as common grackles (*Quiscalus quiscula*) and house sparrows (*Passer domesticus*) develop high viremia with lower mortality rates [90]. American robins (*Turdus migratorius*) and house finches (*Carpodacus mexicanus*) are considered important amplifying hosts in different regions of the U.S. [91]. In addition to birds, at least 30 other vertebrate species, including reptiles, amphibians, and mammals, are susceptible to WNV infection. However, only a few nonavian vertebrates, including brown lemurs (*Lemur fulvus*), lake frogs (*Rana rinibunda*), hamsters, fox squirrels (*Sciurus niger*), eastern gray squirrels (*Sciurus carolinensis*), eastern cottontail rabbits (*Sylvilagus floridanus*), and eastern chipmunks (*Tamias striatus*) have been reported to develop viremia levels expected to support vector transmission [89, 92–98]. Humans and horses may suffer serious disease or death from WNV infection but are considered incidental hosts which do not participate in the WNV lifecycle because they do not develop sufficient viremia to infect mosquito vectors (reviewed in [89]).

Although transmission between hosts by mosquitoes is by far the most common route of transmission, WNV can also be transmitted directly if infected animals or mosquitoes are consumed by susceptible hosts or if susceptible birds come in close contact with cloacal or oral fluids from other birds with high WNV viremia [89].

WNV can also be transmitted between humans by blood transfusion, organ transplantation, transplacental transmission, and via breast milk [99–103]. Although blood donations in the United States have been screened for WNV by nucleic acid testing since 2003, thirteen instances of transfusion-associated transmission have occurred, most recently in 2012 [104–107].

### 4.2. Vectors

Mosquitoes are the vector for natural transmission of WNV. After a mosquito feeds on an infected competent host, the arbovirus replicates within the mosquito and can then be transmitted to a susceptible host through salivary gland secretions (Figure 4). Compared to related arboviruses such as dengue virus and yellow fever virus, WNV can be transmitted by a variety of mosquitoes with different host-feeding preferences with up to 45 species and 8 genera reported positive in the U.S. between 2004 and 2008 [108]. However, not all mosquito species reported as WNV-positive are competent vectors of WNV, and not all species that are transmission-competent in the laboratory will play a role in natural transmission [109].

Mosquitoes that feed on both birds and mammals are referred to as bridge vectors for WNV because they act as a “bridge” between an infected reservoir (birds) and mammalian incidental hosts which do not develop sufficiently high viremia to support transmission to mosquitoes [110, 111]. Ornithophilic mosquitoes play an important role in maintaining and amplifying transmission among birds but typically do not play a role in transmission to humans [109, 111]. Mosquitoes of the genus* Culex* have been reported as the most important bridge vectors in the United States, with* Cx. pipiens* as the dominant bridge vector in the northeastern, north-central, and mid-Atlantic United States,* Cx. quinquefasciatus* in the south and southwest, and* Cx. tarsalis* in the west [110, 112].* Culex* spp. mosquitoes have also been implicated in transmission in Europe, Australia, and South Africa [113–116]. Mosquitoes of the genus* Aedes*, the transmission vector for related flaviviruses, may also serve as important bridge vectors [108, 109]. While experimental transmission of WNV by ticks has been demonstrated, a role for ticks in natural transmission and maintenance of WNV has not been determined [117–121].

## 5. Epidemiology and Clinical Outcomes of Human Infections

Most human infections with WNV (~80%) are asymptomatic, and symptomatic infections may vary from flu-like malaise to serious neuroinvasive diseases, for which there is no specific treatment. Fewer than 1% of human infections progress to severe disease, for which the most frequently reported risk factors include advanced age, immune suppression, and chronic medical conditions including, but are not limited to, hypertension, diabetes, and chronic renal failure [122–131]. In 2002, out of more than 4000 cases reported to the CDC, 150 cases were in patients of age 19 or younger. The youngest fatality was a 19-year-old patient, and the median age among fatal cases was 78 years [132]. In the outbreak of 2003, at least 31 cases of WNV encephalitis and 79 cases of WNV meningitis occurred among children and adolescents; however, there were no fatalities caused by WNV disease in children or adolescents [132].

Seroepidemiological studies suggested that one in four to one in five (20–25%) WNV-infected individuals develops mild illness [133, 134] and one person in 150 (0.67%) develops WNND [135]. Subsequent epidemiological studies using asymptomatic infection data obtained from nucleic acid testing to screen blood donations combined with the reported cases to the CDC concluded that one in 244 to one in 353 infections will progress to WNND [123, 136]. These findings suggested that more asymptomatic WNV infections could be identified when prospective studies focused on healthy populations such as blood donors [123, 136]. A serosurvey following lineage 2 WNV infections in Greece in 2010 yielded estimates of one in 124 to one in 141 infections leading to WNND, with approximately 18% of infected individuals showing symptoms [137].

Among WNND patients, 50–71% develop WN encephalitis, 15–35% develop meningitis, and 3–19% develop acute flaccid paralysis [126, 128, 138–145]. Severe cases have fatality rates ranging from 3% to 19% in encephalitis cases [126, 128, 130, 139, 140, 142–144, 146, 147]. Loeb et al. reported that physical and mental impairment resolve in about a year, but patients with preexisting comorbid conditions take longer to recover [148]. However, other studies of patients infected with WNV have noted physical symptoms and/or cognitive deficits persisting over a year after infection in more than half of WNND cases [141, 149]. Persistence of WNV symptoms >6 months was reported most often in patients with WNND, hypertension, and diabetes [150]. WNND has also been reported as a risk factor for development of chronic kidney disease in a long-term follow-up study of WNV patients [151].

Although fewer cases are available from which to conduct a detailed study, it is thought that both the risk of illness and the risk of neuroinvasive infection are lower from the WNV Kunjin subtype (lineage 1b), which circulates in Australia [152]. Until recently, viruses in lineage 2 were not believed to cause WNND in humans. However, outbreaks of lineage 2 WNV strains in the past 10 years in Russia and Greece have caused WNND and death, with case fatality rates similar to those observed previously for lineage 1 WNV [124, 147, 153]. WNND caused by WNV lineage 2 in horses and humans in South Africa has also been reported [79, 154–156].

## 6. WNV in Africa and the Middle East

West Nile virus was first observed in Africa, in the West Nile district of Uganda, 1937 [10], and thus had been known in the Old World for over 60 years before it crossed the Atlantic. Though it was first isolated from a febrile human case, WNV was observed to cause relatively mild disease in humans and no deaths were reported from the early epidemics studied [10]. While the introduction and progress of WNV through the New World could be studied as it occurred, epidemics of WNV were believed to have occurred throughout much of Africa, the Middle East, and south Asia before clinical WNV was observed in humans in those areas. A 1939-1940 serosurvey found widespread human seropositivity for WNV, determined by comparison of neutralization titers for WNV, SLEV, and JEV, in Uganda, Sudan, the current Democratic Republic of the Congo, and Kenya, with seropositivity over 50% in some localities [157]. Seropositivity was also found in western Nigeria, in samples collected in 1951 and 1955 [158]. In South Africa, seropositivity in humans who had not traveled, monkeys, domestic animals, and juvenile birds was demonstrated in samples collected in 1954 [159]. Therefore, the past presence of WNV had been demonstrated over a wide geographic range in Africa before clinical infections were observed in most locations.

Following its first isolation in 1937, WNV was not isolated again until 1950. During a serosurvey conducted of 251 individuals, mostly children, living in Cairo, isolates were generated from the serum of three children, only one of whom had been diagnosed with a fever [160]. The same serosurvey noted that more than 70% of the study participants aged 4 and above carried neutralizing and complement-fixing antibodies to WNV and that over 50% of infants carried maternal antibodies against WNV, indicating that WNV infection was widespread among the population and that most individuals were infected as young children [160]. A subsequent serosurvey in northeastern Egypt demonstrated widespread seropositivity of adults and children at multiple locations in the Nile Valley excluding one coastal location, indicating that WNV was not only endemic in Egypt but frequently transmitted [161]. WNV was also isolated from* Culex* spp. mosquitoes in Egypt in 1952 [162].

The first known isolation of West Nile virus in Israel was from a febrile child in 1951, as part of an outbreak that occurred on an agricultural settlement near Haifa [2]. Morbidity in children in this outbreak was substantially higher than in adults, and subsequent outbreaks in Israel in 1952 and 1953 occurring primarily in adolescents and adults were also identified as WNV, on the basis of isolation of the virus from human cases and serology from human cases and chickens [2, 6, 7]. However, WNV is believed to have been present in Israel prior to these isolations, because prior outbreaks between 1942 and 1950 were observed to have been similar clinically and epidemiologically to the ones in 1951 and 1952 [6]. Illnesses in these cases were generally self-limiting with recovery slower in adults than children [2, 6]. WNV fever was described as a “benign specific short-term fever occurring in epidemic form” and was believed to cause only mild neuroinvasive cases [7]. The first fatalities due to WNND were reported in a cluster of elderly patients in 1957; however, overall, neurological involvement in WNV cases was considered unusual [4, 10, 11, 16]. In 2000, the first WNV outbreak in Israel since 1980 was reported, with 417 serologically confirmed cases and 35 deaths [16]. Viral isolates from this outbreak were most closely related to isolates from the 1996 Romanian and 1999 Russian outbreaks [163, 164]. Since then, Israel has experienced regular annual summertime outbreaks of varying size, similar to those observed in the United States [126, 165–167].

Human seropositivity for WNV in Turkey was documented in the 1970s, and again beginning in the mid-2000s [168–172]. An outbreak of WNV occurred in Turkey in 2010-11, concurrent with other outbreaks in the Mediterranean region, causing 47 cases including 40 WNND cases and 10 fatalities [173].

Seropositivity for WNV was also reported in Iran in the 1970s [174]. A 2008-2009 survey of patients with fever and loss of consciousness identified 3 cases which were positive by RT-PCR and 6 more that were positive by IgG [175]. A study of horses conducted from 2008-2009 identified IgM-positive animals and seroprevalences up to 88% in some regions, with the highest activity in western and southern provinces [176]. Serologic evidence for infection has also been found in Jordan and Lebanon although no human cases have been reported from those countries [177–180].

WNV continued to circulate in northern and sub-Saharan Africa throughout the late 20th and early 21st century, causing outbreaks in Algeria, Morocco, Tunisia, the Democratic Republic of the Congo, and South Africa, along with sporadic cases and seropositivity in humans and/or horses distributed throughout the continent [165, 181–184]. Active transmission has continued in northern Africa, with outbreaks reported in Morocco in 2010 and Tunisia in 2012 and ongoing sporadic transmissions in Egypt and Algeria [165, 183, 185–189].

The regular pattern of infection in South Africa prior to 1974 was sporadic, relatively mild human infections and epizootics, with epidemics in humans occurring in 1974 and 1984 (reviewed in [8]). The relative nonpathogenicity of human and equine infections in South Africa had been attributed to reduced pathogenicity of lineage 2 WNV strains; however, later reports of WNND caused by lineage 2 WNV infections in South Africa suggested that the full clinical extent of WNV infection in earlier epidemics may not have been recognized [79, 156]. In 2010, the first case of lineage 1 WNV occurring in South Africa caused the death of a pregnant mare [190]. Infections caused by lineage 2 in Madagascar have also generally been considered mild to inapparent; one fatal case of WNND originated in Madagascar in 2011, although it was speculated that the patient had a deficient antibody response [191].

Recent reports have indicated ongoing transmission in other regions of sub-Saharan Africa. Eleven cases of acute febrile illness were caused by WNV in Guinea in 2006 [192]. A 2009 seroprevalence study in Ghana indicated that WNV is endemic, with most WNV cases occurring in childhood [193]. A fatality due to WNND was reported in Gabon in 2009 [194]. A study in Nigeria demonstrated that 25% of tested febrile patients, many of whom were infected with* Plasmodium falciparum* or* Salmonella* Typhi, were seropositive for WNV, suggesting that WNV infection in this region may be mistaken for these diagnoses or for other cocirculating arboviruses [195]. Seroconversion of sentinel chickens was observed in Senegal in 2009 [196]. In eastern Africa, human infections and mosquitoes positive for WNV lineage 2 were reported in Djibouti from 2010-2011 [197]. Recent positivity for WNV in Kenya has also been reported in ticks collected from 2010–2012 and mosquitoes from 2007–2011 [121, 198].

## 7. WNV in Southern and Eastern Asia, Australia, and Oceania

In the early 1950s and 1960s, seropositivity for WNV was demonstrated throughout India and as far east as Myanmar [199, 200]. Sporadic cases were documented in India throughout the 1970s–2000s [3]. Most sequenced isolates from India separate into a distinct lineage referred to as lineage 5 or lineage 1C, although lineage 1A isolates have also been reported [3, 72, 86, 201]. Although WNV had previously been shown to cause neurological disease, the first pediatric fatalities from WNND were reported in India, where three children died of WNV encephalitis in 1981 [5]. Pediatric WNND cases have been frequently reported in Indian WNV outbreaks, in contrast to North American and European WNV outbreaks, in which pediatric cases are relatively infrequent [3, 5, 202–204].

Recent reports on WNV in India have included ongoing isolation and sequencing of both WNV lineages 1A and 5, as well as cocirculation and possibly coinfection with JEV in 2006 in areas of northeastern India where both WNV and JEV are endemic [86, 201, 202, 204, 205]. The 2010 outbreak of lineage 1 WNV in Tamil Nadu state was associated with ocular disease, an infrequently reported WNV complication [201]. The recent lineage 5 isolates reported from northeastern India were more neuroinvasive and pathogenic in mice than Indian isolates from 1982 and earlier [86]. Cases of WNND have also been reported from Pakistan along with human seroprevalences ranging from 12 to 54% [206–209].

Isolations of WNV have been reported in Malaysia and Cambodia, and seropositivity for WNV was also noted in Myanmar, Thailand, and the Philippines [88, 200, 210–212]. Lineage 2 WNV was sequenced from an acute febrile specimen collected in 2004 in Indonesia [81]. Recently, WNV has also been isolated from clinical specimens collected in Nepal from 2009-2010; sequenced fragments of both isolates showed homology primarily to lineage 1 viruses, but one fragment of each was more similar to lineage 2 than to lineage 1 [213].

In China, seropositivity for WNV was first reported in birds from Yunnan province in 1988 [214, 215]. The first confirmed human cases of WNV in China were reported in 2013 but occurred during a 2004 outbreak of fever and neurological disease in Xinjiang province in northwestern China, in which diagnosis was delayed due to antibody cross-reactivity with Japanese encephalitis virus [214]. Seropositivity for WNV in Shanghai was reported in 14.9% of cats and 4.9% of dogs tested in 2010, as well as in captive resident birds from 2009-2010, but no human cases have been reported from southern or eastern China [216, 217]. In South Korea, antibody against WNV was detected in 5/1531 bird specimens in a 2009 study, but no resident birds were seropositive [218].

Kunjin virus, which was originally considered to be a closely related virus but is now considered a subspecies of WNV lineage I, was first isolated in Australia in 1960 [219, 220]. Symptoms of WNV/Kunjin in Australia are considered relatively mild, with infrequent WNND and no deaths reported [115, 221]. WNV/Kunjin has continued to cause intermittent cases of equine and human disease in Australia, primarily in the northwest, where it frequently cocirculates with the related flavivirus Murray Valley encephalitis virus (MVEV) [221]. In 2011, an outbreak of WNV (co-circulating with MVEV) in horses in southeastern Australia was attributed to a strain of WNV designated WNV_NSW2011_, which was closely related to Kunjin virus, but carried two amino acid changes previously associated with increased virulence in North American WNV NY99 strains [222–224]. These changes rendered WNV_NSW2011_ significantly more neuroinvasive than previously observed Australian strains. However, no human cases were reported from this outbreak, and a serosurvey in Victoria showed little evidence of recent human WNV infection [225].

## 8. WNV in Europe

The presence of WNV was first discovered in Europe in 1958 in Albania with detection of neutralizing antibodies in human sera [226, 227]. The first documented outbreak of WNV occurred in southern France in 1962-1963, causing WNND in both humans and horses [1, 9]. Following that outbreak, no further WNND cases in humans were reported until 1985, although virus activity in the region has been confirmed on multiple occasions. The virus was isolated from mosquitoes in Portugal and the Czech Republic, migrating birds in Slovakia, and western Ukraine, and ticks in Hungary and the Moldavia region [227–231]. WNV was also sporadically detected in serological surveys of humans, migratory birds, and domestic animals in the countries throughout southern and eastern Europe and the Mediterranean basin, although the virus was not considered a public health concern during that time due to the absence of reported WNND [227, 232, 233].

However, the situation has changed dramatically in the last three decades with a series of symptomatic WNV outbreaks in several European countries. Human WNND cases were first observed in western Ukraine in 1985, followed by a period of relative silence and two major epidemics: in Romania in 1996 and in Russia in 1999 [14, 15, 232]. During the outbreak in Romania, 835 patients were hospitalized with neurological symptoms and 343 were confirmed to be WNV-positive. The epidemic caused 17 deaths [15]. The mortality rate was even higher in Russia: out of 826 patients who presented with symptoms, 183 tested positive in serological essays, and 40 died of acute aseptic meningoencephalitis [14].

Other notable outbreaks with human cases from 2000–2009 include the reemergence of WNV in France in 2000–2003, Italy in 2008-2009, and Hungary in 2008 [232, 234, 235]. In France in 2000, WNND cases were confirmed in 76 horses, and 21 of them died; interestingly, the same region of the country was affected as in the outbreak of 1962 [236]. WNND cases described in 2003 involved horses and humans [237]. A subsequent serologic survey in horses suggested the possibility of persistent WNV circulation in the area [238]. The 2008 outbreak in Italy was preceded by a 1998 event with 14 encephalitic equine cases and 4 asymptomatic cases in humans; a retrospective study revealed a 38% seroprevalence rate in horses in the region [239, 240]. In 2008-2009, both equine and human WNND cases were reported [241]. In Hungary, the sudden spread of the virus in 2008 caused 12 equine and 22 human neuroinvasive cases [234]. Following the large outbreak in 1999, Russia experienced annual summer transmissions with sporadic outbreaks primarily in the south [242]. The three most affected regions were Astrakhan, Rostov, and Volgograd provinces with outbreaks in 2007, 2010, and 2012, although recently the range of the virus has apparently expanded, with cases reported further north- and eastward in several provinces including southern parts of Siberia [166].

With the outbreaks becoming more frequent and sporadic cases surfacing all over Europe, enhanced surveillance programs were established in many European countries [232, 243]. One such program was instrumental in promptly identifying WNV cases during the largest recorded outbreak in Italy in 2012, where simultaneous circulation of both WNV lineage 1 and lineage 2 was documented [244–247]. From 2010–2013, human WNV cases were reported in Austria, Bosnia and Herzegovina, Croatia, Greece, Hungary, Italy, Kosovo, the Former Yugoslav Republic of Macedonia, Montenegro, the Russian Federation, Serbia, Spain, and Ukraine [165, 248–253]. Greece and Russia experienced high WNV activity each year from 2010–2013, and 302 cases were reported from Serbia in 2013 [165, 166]. Additionally, the presence of WNV was confirmed in the Czech Republic, Portugal, and other countries where it had not previously been identified, with most of the reports suggesting seasonal introduction by multiple routes and continuous low-level WNV circulation in Europe [254, 255].

Notably, prior to 2008, lineage 1 viruses were responsible for severe WNND cases in humans in Europe, and lineage 2 viruses were only reported in sub-Saharan Africa and Madagascar until 2004. However, the cases in Hungary were caused by lineage 2 WNV, with subsequent spread into Austria, Italy, Russia, Greece, Serbia, and Croatia [85, 248, 255–260]. The strain isolated from the 2010 Greek outbreak, WNV Nea-Santa-Greece 2010, was shown to carry a mutation previously associated with increased virulence in corvids in lineage 1 WNV strains [261, 262].

Countries with established surveillance programs and no reported clinical cases so far include the U.K., Germany, and Switzerland [263–265]. One serologic study in the U.K. identified WNV-seropositive wild birds, although subsequent studies have not found evidence of WNV circulation in birds [266–269]. In Germany and Poland, seroprevalence in birds was relatively low [264, 270].

## 9. WNV in the United States

WNV infection is a major public health concern in the United States, where the virus has become endemic causing recurring outbreaks for 14 consecutive years. The initial outbreak in the U.S. resulted in 62 reported cases, including 59 WNND cases and 7 deaths; however, estimates based on serosurveillance suggest that 2.6% of the population near the outbreak epicenter in New York City was affected in that outbreak [133]. The WNV strain associated with the U.S. outbreak, designated WNV NY99, was a lineage 1 strain closely related to an isolate from the outbreak in Israel in 1998, and both the U.S. and Israeli strains were related to a Tunisian isolate from 1997 [70, 71, 77, 271]. By the summer of 2000, the virus had also caused human disease in the states of New Jersey and Connecticut with a total of 21 cases reported including 19 WNND cases that resulted in 2 deaths [272].

In contrast to the historically observed pattern of outbreaks in Europe and Africa between the 50s and the 90s, in which epidemics were followed by years of inactivity, WNV continued to spread in the U.S. following its introduction. In the summer of 2001, the virus was found in 10 states with 66 total reported cases (64 WNND) and 9 deaths. In 2002, 40 states reported a total of 4,156 WNV human cases to the CDC, with 70.9% (2,946) classified as WNND resulting in 284 fatalities. Possibly due to increased awareness in the medical community through an outreach program by the HHS, in 2003 the total number of reported cases increased 42% to 9,862; however the number of WNND cases declined to 2,866, which represented 29% of reported cases. By 2004, WNV had been detected in all of the contiguous 48 states and was considered endemic. Another large outbreak occurred in 2006, with 177 deaths and 1,495 WNND cases, out of a total of 4,260 cases. Coincident with the 2002-2003 outbreaks, a new viral genotype known as WN02 replaced the original viral genotype NY99; the new genotype was observed to disseminate more efficiently in North American* Culex pipiens* and* Culex tarsalis* mosquitoes than the NY99 genotype [273–276].

**Figure 5:**
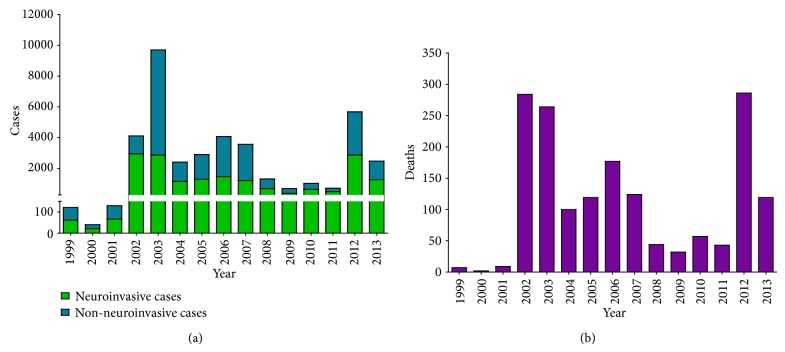
(a) Neuroinvasive and nonneuroinvasive cases of WNV in the United States reported to the CDC, 1999–2013. (b) Deaths from WNV infection in the United States reported to the CDC, 1999–2013.

The intensity of WNV activity in the U.S. was very high between 2002 and 2007, with over 1,000 WNND cases per year. A decline in the number of cases began in 2008, and comparatively low activity continued through 2011 when only 712 total WNV cases were reported. In 2012, however, another large outbreak of WNV occurred in the U.S., causing 2,873 WNND cases and the most deaths [277] ever reported in a single WNV season. Activity in the U.S. in 2013 was moderate, with 1,267 WNND cases and 119 deaths in 2013 reported as of May 9, 2014. From 1999–2013, there have been a total of 39,557 reported cases of WNV in the U.S. of which 17,381 were WNND, resulting in 1,667 deaths, an average of 111.1 deaths/year [278] (Figure 5).

Based on epidemiological estimates that for each case of WNND there are 150 to 350 human infections, 2.6 to 6.1 million people in the United States have been infected with WNV over the past 14 years. Through 2010, it was estimated that 1.1% of the U.S. population had been infected by WNV, with the highest incidence in the state of South Dakota (13.3%) [123, 279]. Because most WNV infections are asymptomatic or mild, many human infections may not be recognized, and there may be significant underreporting of milder symptomatic cases [133, 279].

## 10. WNV in Other Parts of North America

**Figure 6:**
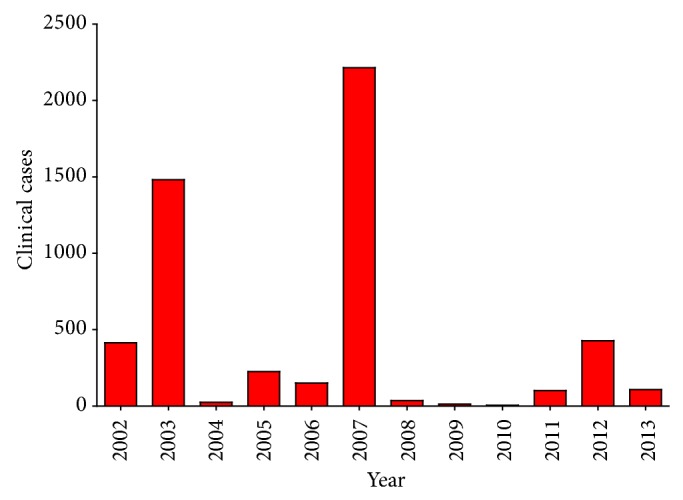
Total clinical WNV cases in Canada reported to the Public Health Agency of Canada, 2002–2013.

In 2001, WNV was first detected in 128 dead birds and 9 mosquito pools in Ontario, Canada [280]. Human cases in Canada were first reported in 2002, with 394 in Ontario and 20 in Quebec [281]. In 2003, WNV spread westward to Manitoba, Saskatchewan, and Alberta, but did not reach British Columbia until 2009 [281, 282]. Since 2002, Canada has experienced annual summer outbreaks similar to those in the United States, with the largest outbreaks occurring in 2003 (1,481 cases), 2007 (2,215 cases), and 2012 (428 cases) [281, 283] (Figure 6).

The first WNV activity in the Caribbean was a human WNV encephalitis case reported at the end of 2001 in the Cayman Islands [284]. Most Caribbean WNV activity for the next few years was limited to observations of seropositivity in birds and horses. In 2002, WNV activity was observed in migratory and resident birds in Jamaica and the Dominican Republic, and in horses in Guadeloupe. An avian serosurvey in Jamaica, Puerto Rico, and Mexico in spring 2002 reported detection of specific neutralizing antibodies for WNV in 11 resident species from Jamaica only [285]. In the Dominican Republic, a seroepidemiological study performed in birds sampled in November 2002 on the eastern side of the country showed anti-WNV antibodies in 15% (5/33) of resident birds [286]. By 2003, WNV seropositivity had also been detected in resident birds on the northwestern side of the Dominican Republic [277]. A serosurvey of 360 healthy horses in Guadeloupe showed an increase in prevalence of IgG antibodies to WNV from 8.8% in June 2002 to 50% in January 2003, indicating a high incidence of WNV infections in horses within that 6-month period [287].

The second Caribbean human WNV encephalitis case occurred in the Bahamas in July 2003 [288]. WNV was detected in Trinidad in October, 2004, in a serosurvey of 60 horses and 40 birds, with reported seropositivity of 3% and 5%, respectively [288, 289]. In Cuba, WNV infection was confirmed by serologic assays in 4 asymptomatic horses and 3 humans with encephalitis in 2003 and 2004 [290]. A 2004 serosurvey of over 1900 resident and migrant birds in Puerto Rico and Cuba found 10 WNV-positive birds (9 migrant, 1 resident) in Puerto Rico and 3 birds (1 migrant, 2 resident) in Cuba [291]. Three seropositive horses were observed in 2004-2005 in Puerto Rico, followed by detection of WNV in three blood donors in 2007 [292]. A 2007 isolate from Puerto Rico carried the mutation V159A in the envelope gene, which is characteristic of the WN02 genotype which replaced the original NY99 genotype in the U.S. [293]. Two further WNV human cases were detected in Haiti during surveillance of febrile patients following Hurricane Jeanne in 2004 [294].

In 2002, WNV appeared in Mexico, with reports of encephalitis-like illness in horses in different areas, concurrent with reports of WNV encephalitis outbreaks in horses along the Texas border in the states of Coahuila, Tamaulipas, and Chihuahua [295]. Mexico has reported low numbers of cases in humans, horses, and birds, primarily from the northern border with the United States. The first confirmed autochthonous human case of WNV in Mexico was reported in 2004 [296]. A fatal human case in 2009 was reported in a 40-year-old man who had mild symptoms for several weeks then progressed to neurological disease, coma, and death [297].

A surveillance study in Mexico found relatively low levels of WNV transmission and disease, which were attributed to multiple factors including the interactions of amplifying hosts, vectors, and circulating virus strains, in combination with climate, habitat, and circulation of interfering flaviviruses [298]. A Mexican isolate from 2003 was shown to have reduced pathogenicity for mice, crows, and sparrows, which may also have contributed to a reduced presence of WNV in Mexico [299, 300].

In El Salvador, an investigation of outbreaks from 2003 revealed that 25% (18/73) of equine specimens tested had antibodies to WNV and were confirmed by plaque reduction neutralization tests (PRNT) [301]. In October 2003, WNV was also identified in horses in Belize [289].

A 2003-2004 serosurvey conducted at multiple locations in Guatemala detected 9 horses positive for WNV [302]. Seropositivity in horses in Costa Rica was also reported from a 2004 serosurvey [303]. The only human case of WNV from Nicaragua was reported in a Spanish missionary who became ill in the summer of 2006 while living in Nicaragua and was subsequently transferred to Spain for treatment, where he was diagnosed [18].

## 11. WNV in South America

The first detection of WNV in South America was in an autumn 2004 epidemiological survey of horses which had not been vaccinated against WNV or traveled outside of Córdoba and Sucre in the Caribbean region of Colombia [304, 305].

WNV was next reported in northern Argentina, where WNV was isolated from the brains of 3 horses that died from encephalitis in February 2006 [306]. A later report showed that resident birds in Córdoba, Chaco, and Tucumán provinces had antibodies to WNV as early as January 2005, and seroconversions were observed in three birds between January and March 2005 [307]. In December 2006, health authorities reported 4 human cases, 1 case in the city of Marcos Juarez in Córdoba province and 3 additional cases in Chaco province [17]. The case in Córdoba occurred in March of that year in a 58-year-old man who had not traveled outside the country in recent years, suggesting that the disease was contracted locally [17].

In 2006, WNV was also reported in birds and horses in Venezuela, in a seroepidemiological study with PRNT confirmation [308]. WNV was detected in Brazil in a study performed on samples collected in 2009 from mosquitoes, horses, and caimans from the Pantanal region of Central-West Brazil, in which a total of 5 out of 168 horse specimens tested positive for WNV, using a flavivirus-specific epitope-blocking enzyme-linked immunosorbent assay with confirmation of reactive specimens by PRNT [309]. Further studies performed with specimens from the Pantanal region, where WNV cocirculates with multiple other flaviviruses, have found WNV seropositivity confirmed by PRNT in horse and chicken samples collected in 2009 and later [310–312]. WNV seropositivity was also reported in an equine sample collected in 2009 in Paraiba state in northeastern Brazil [313]. However, WNV has not yet been associated with human or equine illness in Brazil [314].

A study conducted on a subset of 20,880 samples from individuals with acute febrile illnesses from Bolivia, Paraguay, Ecuador, and Peru from 2000–2007 identified at least one patient with PRNT-confirmed seropositivity for WNV; however, no virus was isolated, and the number and location of WNV-positive patients were not given [315]. A 2011 serosurvey of horses in Bolivia found PRNT-confirmed seropositivity for WNV, although no horses were IgM-positive, indicating that WNV had circulated in the area prior to 2011 [316].

Interestingly, sequences obtained from Colombian viral isolates in 2008 were most closely related to 2001 Louisiana, U.S. sequences of the NY99 genotype, suggesting that the WN02 genotype which replaced NY99 in the U.S. had not progressed southward over that time period [317].

## 12. Conclusion

Since its discovery in 1937, West Nile virus has spread beyond its original known geographic range and caused human disease on every continent except Antarctica. It is now the most widespread cause of arboviral neurological disease in the world. With no vaccine available to date and limited treatment options, transmission via organ donation and blood transfusion also poses a risk.

While expansion of WNV into Central America, South America, and the Caribbean has been marked by relatively few human WNND cases and recovery of attenuated viruses, characterization of strains isolated from recent outbreaks in Greece, Australia, and India shows increased virulence in tissue culture and/or animal models [86, 222, 262, 300]. However, none of the observed changes has been directly correlated to virulence in human infections.

Recent large outbreaks of human WNND in Europe and North America, as well as ongoing transmission in the Middle East, Africa, and Asia, illustrate the need for continued surveillance and preventative measures. The risk for transmission and outbreaks remains high in the many parts of the world with suitable mosquito vectors.

## Supplementary Material

The WNV statuses (human cases, non-human cases, or no positivity reported) shown in Figure 1 were assigned based on a review of the available literature. Selected references supporting the WNV statuses shown in Figure 1 are presented in the Supplementary Materials.
