# Supplementary material for: The Global Ecology and Epidemiology of West Nile Virus
Source: Biomed Res Int. 2015 Mar 19;2015:376230. doi: 10.1155/2015/376230 (PMC4383390; doi:10.1155/2015/376230)
Supplement: Supplementary file 1 — The WNV statuses (human cases, non-human cases, or no positivity reported) shown in Figure 1 were assigned based on a review of the available literature. Selected references supporting the WNV statuses shown in Figure 1 are presented in the Supplementary Materials. [file 376230.f1.pdf]

Supplemental table 1.

Country or Territory	WNV reports	Reference
Albania	Human	(226)
Algeria	Human	(165)
Angola	Human	(318)
Argentina	Human	(17)
Armenia	Non-human	(319)
Australia	Human	(219)
Austria	Human	(250)
Azerbaijan	Human	(320)
Bahamas	Human	(288)
Belarus	Human	(321)
Belize	Non-human	(289)
Bolivia	Non-human	(322)
Bosnia and Herzegovina	Human	(165)
Botswana	Human	(323)
Brazil	Non-human	(309)
Bulgaria	Non-human	(324)
Burma (Myanmar)	Human	(200)
Cambodia	Human	(88)
Cameroon	Human	(325)
Canada	Human	(280)
Cayman Islands (UK)	Human	(283)
Central African Republic	Human	(326)
Chad	Non-human	(181)
China	Human	(214)
Colombia	Non-human	(305)
Congo	Non-human	(182)
Congo, Democratic Republic of	Human	(327)
Costa Rica	Non-human	(328)
Croatia	Human	(251)
Cuba	Human	(290)
Cyprus	Non-human	(210)
Czech Republic	Human	(255)
Djibouti	Human	(197)
Dominican Republic	Non-human	(285)
Egypt	Human	(160)
El Salvador	Non-human	(301)
Ethiopia	Human	(329)
Finland	--*	(330)
France	Human	(9)
Gabon	Human	(194)

Gambia	Human	(331)
Germany	Non-human	(264)
Ghana	Human	(332)
Greece	Human	(262)
Guadeloupe	Non-human	(287)
Guatemala	Non-human	(302)
Guinea	Human	(192)
<u>Haiti</u>	Human	(294)
<u>Hungary</u>	Human	(234)
<u>India</u>	Human	(3)
<u>Indonesia</u>	Human	(333)
<u>Iran</u>	Human	(174)
<u>Israel</u>	Human	(2)
<u>Italy</u>	Human	(241)
<u>Ivory Coast</u>	Non-human	(334)
<u>Jamaica</u>	Non-human	(335)
<u>Japan</u>	--*	(336)
<u>Jordan</u>	Human	(177)
<u>Kenya</u>	Human	(327)
<u>Korea, South</u>	--*	(218)
Kosovo	Human	(165)
Lebanon	Human	(179)
Macedonia	Human	(337)
Madagascar	Human	(191)
Malaysia	Human	(211)
Mauritania	Non-human	(338)
Mauritius	Human	(339)
Mexico	Human	(296)
Montenegro	Human	(188)
Morocco	Human	(186)
Namibia	Human	(340)
Nepal	Human	(213)
Nicaragua	Human	(18)
Nigeria	Human	(158)
Pakistan	Human	(341)
Philippines	Human	(210)
Poland	Non-human	(270)
Portugal	Human	(254)
Puerto Rico (US)	Human	(342)
Romania	Human	(15)
Russian Federation	Human	(14)
Rwanda	--**	(182)
Senegal	Non-human	(181)

Serbia	Human	(252)
Slovakia	Non-human	(229)
Somalia	Human	(343)
South Africa	Human	(159)
Spain	Human	(249)
Sri Lanka	Non-human	(344)
Sudan	Human	(327)
Sweden	--*	(345)
Tajikistan	Non-human	(346)
Tanzania	Human	(347)
Thailand	Human	(348)
Trinidad and Tobago	Non-human	(289)
Tunisia	Human	(183)
Turkey	Human	(168)
Uganda	Human	(10)
Ukraine	Human	(232)
United Kingdom	Non-human	(267)
United States	Human	(133)
Uzbekistan	Human	(349)
Venezuela	Non-human	(308)
Zambia	Non-human	(182)

* In these countries, seropositivity for WNV has been detected only in non-resident birds, which was not considered indicative of local transmission.

** Kading et al reported seropositivity in gorillas living near the border of the Democratic Republic of the Congo and Rwanda, which were sampled in the D.R.C., but may have been infected in Rwanda.
